# Differential Expression of HERV-W in Peripheral Blood in Multiple Sclerosis and Healthy Patients in Two Different Ethnic Groups

**DOI:** 10.3389/fphar.2019.01645

**Published:** 2020-01-30

**Authors:** Rachael Tarlinton, Belinda Wang, Elena Morandi, Bruno Gran, Timur Khaiboullin, Ekatarina Martynova, Albert Rizvanov, Svetlana Khaiboullina

**Affiliations:** ^1^School of Veterinary Medicine and Science, University of Nottingham, Loughborough, United Kingdom; ^2^Centre de Physiopathologie de Toulouse Purpan (CPTP), Université de Toulouse, UPS, INSERM, CNRS, Toulouse, France; ^3^Clinical Neurology Research Group, Division of Clinical Neuroscience, University of Nottingham School of Medicine, Nottingham, United Kingdom; ^4^Republican Research and Clinical Center of Neurology and Neurosurgery, Kazan, Russia; ^5^Insitute of Fundamental Medicine and Biology, Kazan Federal University, Kazan, Russia; ^6^Department of Microbiology and Immunology, University of Nevada, Reno, NV, United States

**Keywords:** human endogenous retrovirus W, multiple sclerosis, multiple sclerosis associated retrovirus, human endogenous retrovirus, ethnicity

## Abstract

Overexpression of the Human endogenous retrovirus W (HERV-W) group of inherited retroviruses has been consistently linked with Multiple Sclerosis (MS). However most of the studies on this link have focused on European genetic groups with a very high risk of MS and it is not clear that this relationship holds for all ethnic groups. This study examined *via* qPCR the RNA expression in peripheral blood of HERV-W (the multiple sclerosis associated retrovirus variant MSRV) of MS patients and healthy controls from two ethnic groups with very different risk rates of MS. Population one was derived from the UK with a Northern European genetic background and an MS risk rate of 108/100,000, population two was derived from the republic of Tatarstan, Russian Federation, with a mixed Russian (Eastern European) and Tartar (Turkic or Volga/Urals) population with an MS risk rate of 21-31/100,000. The Russian population displayed a significantly higher basal level of expression of MSRV in both healthy and MS individuals when compared to the British control population with a trend in the Russian population towards higher expression levels in MS patients than healthy patients.

## Introduction

Multiple sclerosis (MS) is a chronic, progressive autoimmune neurological disease. The disease is often debilitating with few effective treatment options currently available. The underlying triggers for the disease are complex and be best summed up as: MS occurring in genetically susceptible individuals who are exposed to the “right” environmental triggers. A familial association is evident in some cases and some distinct restricted genetic groups (such as Sardinians) (Pugliatti et al., 2001) have a high risk. Extensive GWAS studies have demonstrated a compelling support for the HLA allele DRB1*15:01 as the most significant genetic risk factor for MS, with smaller effects attributed to another 11 HLA alleles and 200 non HLA genes (Didonna and Oksenberg, 2017). Other risk factors for MS include gender (women are at higher risk than men), prior Epstein Barr Virus infection, Vitamin D levels (those with lower levels at higher risk).

The prevalence of MS also shows distinct regional variations with the highest rate in Northern European and North American populations. Also, prevalence increases with latitude in countries and this is thought to be linked to UVB exposure and subsequent vitamin D levels. With notable exceptions for certain populations such as the Sardinians and artic peoples with diets very high in fish oils (and vitamin D). Relapses of clinical disease are also associated with season (with a peak in spring) and low vitamin D levels (Harding et al., 2017). Immunoregulatory effects of vitamin D are believed to play role in MS pathogenesis. This assumption is based on data demonstrating distinct seasonal patterns in cytokine profile (and therefore basal inflammatory states) in a variety of disorders including MS (Blatt et al., 2017; Goodin, 2016; Watad et al., 2016).

Endogenous retroviruses (ERVs) are vestiges of retroviral infections from evolutionary history that remain in the host genomes. They make up to 8% of the human genome and are present in all vertebrates (Hayward et al., 2013). While not replicating as infectious viruses in humans they can be expressed in some individuals and are linked to the disease pathogenesis. There is a strong epidemiological link between the expression of the HERV-W retroviral family and Multiple Sclerosis (Morandi et al., 2017a). HERV-W is an attractive candidate for the link between the genetic and environmental factors in MS for a number of reasons. The envelope protein or mRNA of certain HERV-W loci are expressed constitutively in normal human tissues, particularly in the placenta (the syncytin locus), with certain HERV-W RNA sequences isolated from MS patient samples known as multiple sclerosis associated retrovirus (MSRV) (Nellaker et al., 2009; Bhat et al., 2011; Soygur and Moore, 2016). Interestingly HERV-W is upregulated in the brain of MS patients (Morandi et al., 2017a) when compared with healthy controls. Upregulation of HERV-W can be reliably induced by Epstein Barr and other viral infections (Nellaker et al., 2006; Mameli et al., 2012). The HERV-W Env protein also induces a pro-inflammatory responses and neuronal pathology similar to that found in MS (Perron et al., 2013; Morandi et al., 2017b). Intriguingly HIV patients receiving antiretroviral medication are seemingly protected from MS and there have been clinical trials of antiretroviral drugs targeted at HERV-W as MS therapeutics (Morandi et al., 2017b).

Despite this epidemiological data few studies have looked at whether there are differences in HERV-W expression between populations with high and low MS prevalence, or populations of distinctly different genetics, despite this being highlighted as a potential confounding factor in the use of HERV-W expression as a marker of MS disease (Morandi et al., 2017a). This study reports HERV-W expression in MS patients and healthy controls in two distinct populations with very different ethnic makeups and MS risk. The two study locations, the East Midlands in the UK and the Republic of Tatarstan in Russia are at approximately the same latitude but have a different climate, the main differences being in the temperatures of the coldest months (below 0 Celsius in Tatarstan) (Peel et al., 2007). The prevalence of MS is very different between the two sites with a rate of 97-108/100,000 in the UK site (Kingwell et al., 2013) and 21-31/100,000 for the Tatarstan site (Simpson et al., 2011). The ethnic mix of the populations also very different with the East Midlands 85% White British (Northern European), with a sizeable (7%) South Asian minority (Office for National Statistics, 2011) and Tatarstan, 40% Russians (Eastern Europeans), 53% Tatars and large minorities of other groups such as the Chuvashes (3%) (Volga, Ural/Turkic) (Federal State Statistics Service, 2010). Within the Tatarstan population there is a documented lower incidence of MS in the Tatar population when compared with the Russian (Bakhtiiarova and Magzhanov, 2006). Within the East Midlands population there is also a lower incidence of MS in the South Asian compared with the White British population (Albor et al., 2017).

## Materials and Methods

### Human Blood Samples

Blood samples from patients attending Nottingham University Hospitals NHS Trust or the Republican Clinical Neurological Center, Republic of Tatarstan, Russian Federation were collected in PAXgene Blood RNA tubes (Qiagen). The Russian samples were shipped to Nottingham as PAXgene tubes. MS diagnosis was established according to the McDonald criteria (Polman et al., 2011). All patients and health controls (HC) signed informed consent for which ethical approval was obtained. In total 43 UK patients (21 HC and 22 MS) and 25 Russian patients (7 HC and 18 MS) gave samples. Patients and HC age, gender, and clinical status are presented in **Supplementary Table 1**. Ethnicity data where available is presented.

Ethics statement: Informed consent was obtained from each subject according to the clinical and experimental research protocol, approved by the Nottingham Research Ethics Committee 2 (Ref 08/H0408/167) and the Biomedicine Ethic Expert Committee of Republican Clinical Neurological Center, Republic of Tatarstan, Russian Federation (N: 218, 11.15.2012).

### RNA Extraction and cDNA Synthesis

RNA processing for all samples was performed in Nottingham. Total RNA was purified using the PAXgene Blood RNA kit (Qiagen) following the manufacturer’s instructions. The RNA was treated with DNase to remove trace amounts of bound DNA. After the wash steps, RNA was extracted in the elution buffer provided with the kit and stored at -80°C. RNA concentration was determined by measuring the absorbance at 260 nm using NanoDrop ND-100 (Thermo Scientific). For making cDNA, 0.5 μg RNA samples, 2 μl Random hexamers and 1 μl dNTPs mix (stock solution 10 mM) (all Promega) were mixed, followed by a 5 min incubation at 65°C for first strand cDNA synthesis. A master mix containing RNase inhibitor (Promega, UK), DTT (0.1M) and 5X First-Strand Buffer was added, along with Superscript III RT (220 U/μl) (all from Invitrogen, UK). Negative controls replacing the RNA template or the RT with DNase/RNase free H_2_O were included. Samples were incubated as followed: 5 min at 95°C, 60 min at 50°C and 25 min at 70°C. cDNA was stored at -80°C.

### qPCR

Relative quantification was performed using Ubiquitin C (UBC) and Ubiquitin conjugating enzyme E2D2 (UBE2D2) as housekeeping genes as per published literature on stable reference genes for use in PBMC in Multiple Sclerosis (Oturai et al., 2016). For detection of HERV-W nine hundred nM of probe/forward and reverse primer mix “MSRV” (Mameli et al., 2009) forward primer CTTCCAGAATTGAAGCTGTAAAGC, reverse primer GGGTTGTGCAGTTGAGATTTCC, Probe FAM-5′-TTCTTCAAATGGAGCCCCAGATGCAG-3′-TAMRA (Invitrogen custom Taqman assay) UBC and UBE2D2 (TaqMan gene expression assays, Invitrogen, catalogue numbers Hs05002522_g1 and Hs00366152_m1 both FAM-MGB probes) were used. All samples were run in duplicates. Agarose gel electrophoresis of the MSRV primers used in endpoint PCR produced amplicons of the expected size (166 bp) (**Supplementary Figure 1**) QPCR was performed using a BioRad CFX Connect (BioRad) and Faststart Universal probe master (Roche) in 96-well plates with the following cycling conditions: 10 min at 95°C and 40 cycles of 10 s at 95°C followed by 30 s at 60°C. A control sample not subjected to reverse transcription (a “no RT control”) was included in each batch of samples and did not amplify in any instance. Primer efficiencies were calculated from the slope of a standard curve and were 87.2% for HERV-W, 96.8% for UBC and 93.8% for UBE202. Calculation of the relative amounts of HERV-W was performed using the two reference genes with one HC used as a calibrator. Any change in gene expression between patient cohorts and the calibrator HC patient was expressed using the following formula

$$Relative\;gene\;expression=\frac{{\left(E_{GOI}\right)}^{\Delta CTGOI}}{GeoMean[{\left(E_{ref}\right)}^{\Delta CTref}]}$$

Where GOI = HERV-W, ref = UBC, UBE2D2, E = amplification factor (10^[–1/slope]^), ΔCT = Calibrator CT- Sample CT.

## Statistical Analysis

Statistical analysis was performed using Graph Pad Prism 5, Kruskal Wallis test with Dunn’s multiple comparison test *post hoc* testing. Data are presented as medians and interquartile ranges.

## Results

**Figure 1:**
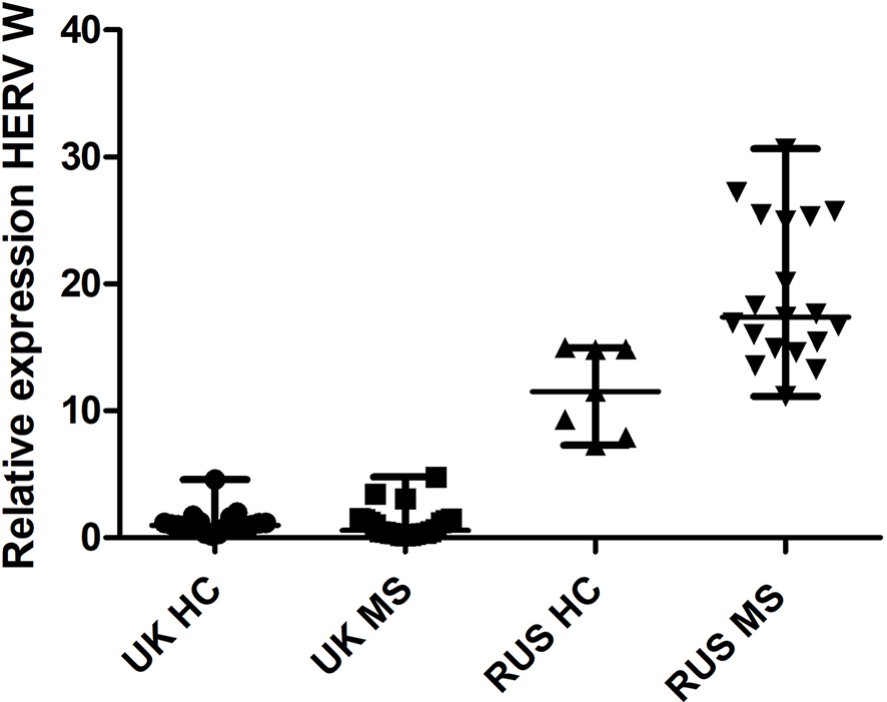
Relative expression of HERV-W against the reference genes UBC and UBE2D2, calibrated against a healthy control (UK) sample. Medians and interquartile ranges are indicated by bars. HC, healthy control, MS, multiple sclerosis, UK, United Kingdom, RUS, Russian (N = 21 UK HC, 22 UK MS, 7 RUS HC, 18 RUS MS), (Medians = 0.98 UK HC, 0.58 UK MS, 11.51 RUS HC, 17.40 RUS MS), Kruskal Wallis P < 0.001, Dunn’s Multiple comparison test P < 0.05 for differences between the Russian and UK cohorts but not between MS and HC. HERV-W, Human endogenous retrovirus W; UBC, Ubiquitin C; UBE2D2, Ubiquitin conjugating enzyme E2D2.

**Figure 2:**
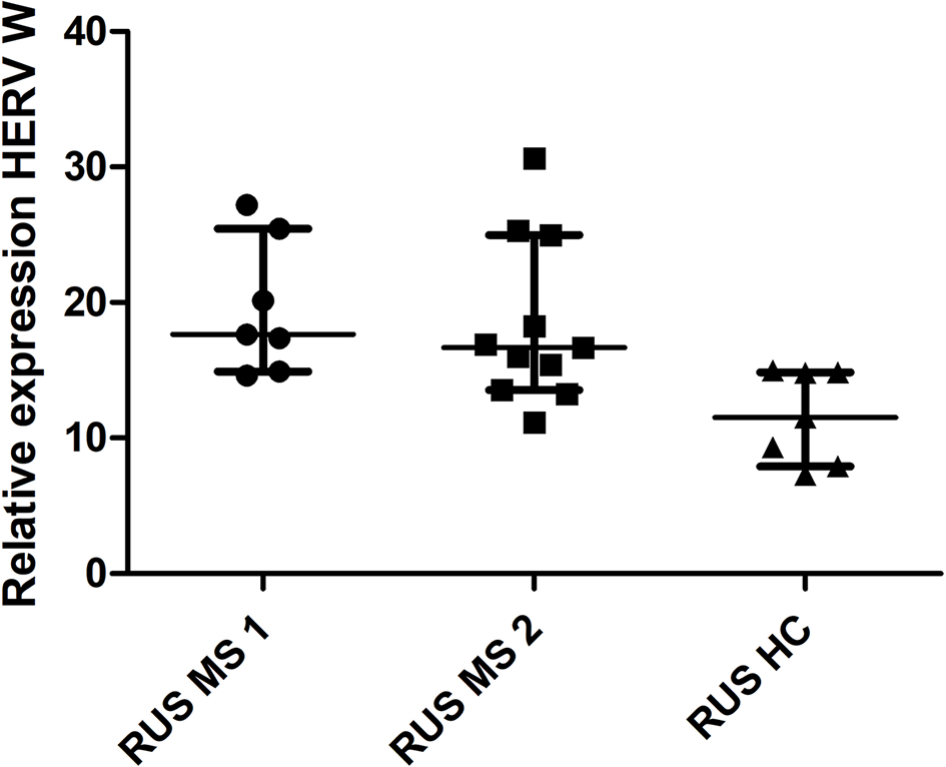
Relative expression of HERV-W against the reference genes UBC and UBE2D2, calibrated against a healthy control (UK) sample. Medians and interquartile ranges are indicated by bars. RUS MS1 = Russian patients on primary presentation (before any disease modifying therapy) RUS MS2 = Russian patients on follow up visits (on a variety of disease modifying therapies) (N = 25, RUS MS1 = 7, RUS MS2 = 11 and RUS HC = 7) (Medians = 17.63 RUS MS1, 16.67 RUS MS2 and 11.51 RUS HC) Kruskal Wallis P = 0.0047, Dunn’s Multiple comparison test P < 0.05 for differences between the RUS MS1 and RUS HC cohorts only. HERV-W, Human endogenous retrovirus W; UBC, Ubiquitin C; UBE2D2, Ubiquitin conjugating enzyme E2D2; HC, healthy control; MS, multiple sclerosis; UK, United Kingdom; RUS, Russian.

There was a significant difference in HERV-W relative expression between the populations (Russian HC, Russian MS, UK HC, and UK MS) (P < 0.0001 Kruskal Wallis) (**Figure 1**). On post-hoc testing (Dunn’s multiple comparison test), there were significant differences (P < 0.05) between the UK and Russian populations but not between the MS and HC within the two populations, though there is a clear trend in the Russian group towards a higher HERV-W expression in the MS patients (**Figure 1**). The Russian patients were divided into those sampled at first presentation (who had not yet had disease modifying therapy) and those seen at follow up visits. There was a significant difference in these cohorts when compared to the Russian healthy controls (P = 0.0095 Kruskal Wallis) (**Figure 2**). On post-hoc testing (Dunn’s multiple comparison test), there were significant differences between the Russian patients on primary presentation and Russian healthy controls but not the other two groups Though a trend, albeit with quite a bit of variability, towards highest HERV-W expression levels in those on primary presentation, an intermediate level of HERV-W expression in those on follow up visits and the lowest levels in healthy controls can be seen (**Figure 2**).

## Conclusions

This study quite clearly shows differences in the expression levels of HERV-W between the UK and Russian populations that are statistically significant. While not statistically significant there is a clear trend in the Russian cohort (but not the UK cohort) towards a higher expression of HERV-W in MS patients than the healthy controls. Other factors such as gender did not display clear differences. The differences between the MS and healthy patients are in line with the reported increase in detection of HERV-W in MS patients in other studies, summarised in the systematic review and meta-analysis in (Morandi et al., 2017a). One caveat is that this study examined HERV-W RNA from whole blood in PaxGene tubes whereas previous studies in the (Morandi et al., 2017a) meta-analysis used a variety of blood derivatives including PBMC, and plasma so the results are not directly comparable. However to our knowledge no previous studies have examined ethnic or population differences in HERV-W expression.

There are a number of potential explanations or confounding factors that could potentially explain these differences. The choice of an unstable housekeeping gene is a common cause of false conclusions from this type of qPCR study, however we performed stability experiments with 5 patients from each cohort (UK MS, UK HC, RUS MS, RUS HC) using a selection of genes from the published work on selection of stable reference genes in PBMC in MS and healthy patients (Oturai et al., 2016) with concordant results to the published work and are confident that this difference is not a technical error. The primer/probe combinations used here not span exon junctions so genomic DNA contamination is a possibility, though at no stage did any of the controls not subjected to reverse transcriptase amplify, indicating that DNA contamination was not present. Even if there were DNA contamination in these samples we do not really expect the copy number of the reference genes or HERV-W loci to vary substantially between individuals so the use of normalisation against the reference genes still should take this into account as a potential confounder. The two populations live at approximately the same latitude removing that as a source of variation in MS risk factors, largely leaving background genetics/ethnicity as the potential explanation of this large difference.

It is also possible that the handling conditions during transport of the Russian samples to the UK, or RNA extractions performed at different time points could have resulted in systematic bias in the HERV-W RNA stability. These loci do not produce virions (which might protect viral RNA while cellular RNA is degraded) and it is hard to image what other processes would have selectively affected the HERV transcripts and not the reference genes. The use of multiple reference genes in relative qPCR studies such as this is specifically designed to balance variations in sample quality and as far as is possible with this study design is the best method currently available to account for this potential confounding factor.

Sample size is also another potential confounding factor and as always these findings will require verification by other groups and with other cohorts of patients. Increasing the sample size is unlikely to change the statistical significance of the differences between the two populations as these are really quite large (there is indeed no overlap in the confidence intervals) and power calculations based on the results in this study give quite trivial numbers (single digits) for confirming the differences between the populations. The differences in the MS patients versus the healthy controls in each population are more subtle and in the case of the British population would require several thousand participants to confirm with confidence, a clearly very different scale of study to the current one.

This study was not designed to stratify differences between stage of disease and HERV-W expression with all the British patients being in the remission phase of the disease and the Russian patients at varying stages with too few patients in each group to adequately compare stage of disease and HERV expression therefore the differences between the Russian patients at first presentation and at later stages of disease need to be taken with some caution. It is possible that stage of disease has also contributed to the differences between the UK and Russian populations, as there is some indication in the Russian cohort that those at first presentation and not currently being treated with disease modifying therapies had higher HERV-W expression, though without longitudinal studies comparing the same patient through relapse and remission this is hard to quantify.

There are several potential explanations for this difference in HERV-W expression; the first is that there may be polymorphisms in the number (presence or absence) or sequence of the HERV-W loci between the two populations. This may affect either the expression profile of the loci or their ability to be detected with the qPCR used in this study. Such polymorphism has recently been reported for HERV-W (Thomas et al., 2018). With the advent of long range deep sequencing technologies capable of distinguishing repetitive loci such as HERVs it may now be possible to determine if such polymorphisms exist between the UK and Russian populations and this is an obvious follow on study to the work presented here. Furthermore, epigenetic dysregulation or cell specific promotor activation of genes other than HERV-W loci (but that may interact with HERV-W expression) are other factors that require exploration in the population differences in HERV expression in the current study.

It is possible that expression of particular defective alleles may even be protective against the expression of deleterious alleles (or exogenous virus) as has been described in other species such as JSRV in sheep (Armezzani et al., 2014). Another possibility is variation in cellular factors that normally suppress HERV expression such as the Trim 5α and APOBEC 3G systems (Fadel and Poeschla, 2011).

Why the expression of a known risk factor for MS is higher in a population with a lower risk of the disease is puzzling, though it seems likely that other genetic or epigenetic factors must be in play here. There has been some work indicating that epitopes of the HERV-W envelope protein could be recognised by the HLA alleles DRB1*1501 and DRB5*0101 (Ramasamy et al., 2017). Therefore, it could be suggested that the failure of HLA allele recognition of the higher levels of HERV-W expression in the RUS MS cohort may account for some of the difference in MS risk. It is however clear that there are population differences in MSRV expression and that this needs to be taken into account when comparing patient cohorts for this risk factor for MS.

## Ethics Statement

Informed consent was obtained from each subject according to the clinical and experimental research protocol, approved by the Nottingham Research Ethics Committee 2 (Ref 08/H0408/167) and the Biomedicine Ethic Expert Committee of Republican Clinical Neurological Center, Republic of Tatarstan, Russian Federation (N: 218, 11.15.2012).

## Author Contributions

RT and AR held the grant funding. RT prepared the manuscript. BW and EMo performed the laboratory work in BG’s and RT’s laboratory. BG, TK, and EMa collected the samples and patient information, SK provided the initial concept and facilitated the Russian sample collection. All authors edited and approved the manuscript.

## Funding

This project was funded by a Royal Society - International Exchanges grant (number IEC\R2\170037). This study was also partially supported by the Russian Government Program of Competitive Growth of Kazan Federal University. AR was supported by state assignments 20.5175.2017/6.7 and 17.9783.2017/8.9 of the Ministry of Science and Higher Education of Russian Federation.

## Conflict of Interest

The authors declare that the research was conducted in the absence of any commercial or financial relationships that could be construed as a potential conflict of interest.
