# Supplementary material for: Differential Expression of HERV-W in Peripheral Blood in Multiple Sclerosis and Healthy Patients in Two Different Ethnic Groups
Source: Front Pharmacol. 2020 Jan 30;10:1645. doi: 10.3389/fphar.2019.01645 (PMC7002920; doi:10.3389/fphar.2019.01645)
Supplement: Supplementary file 2 [file Image_1.pdf]

Supplementary Material

Differential Expression of HERV-W in Peripheral blood in Multiple Sclerosis and Healthy Patients in Two Different Ethnic Groups

Rachael Tarlinton[1*], Belinda Wang[1], Elena Morandi[2], Bruno Gran[3], Timur Khaiboullin[4], Ekatarina Martynova[5] Albert Rizvanov[5], Svetlana Khaiboullina[5,6]

*** Correspondence:** Rachael.tarlinton@nottingham.ac.uk

1 Supplementary Data

2 Supplementary Figures and Tables

.

2.1 Supplementary Figures

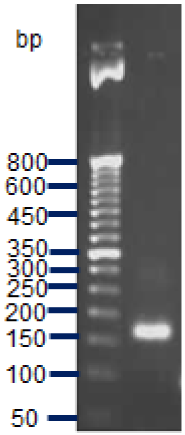

Supplementary Figure 1. DNA gel electrophoresis for validation of gene amplification in RT-PCR. 1 patient sample run on a 2% agarose gel stained with Ethidium Bromide. The first line accommodates a 50 base pairs (bp) ladder. MSRV*env* amplicon size=166bp.

Supplementary Figure 1. The figure legends are required to have the same font as the main text, 12 point normal Times New Roman, single spaced. Please use a single paragraph for each legend and prepare the figures keeping in mind the PDF layout.
